# Thermal micro-insults during routine dental procedures: a forgotten determinant of pulp and dentine fate - a scoping review

**DOI:** 10.3389/fdmed.2026.1917342

**Published:** 2026-10-09

**Authors:** Chinmay D. Bhat, Vandana Sadananda, Gowrish S. Bhat

**Affiliations:** Nitte (Deemed to be University), AB Shetty Memorial Institute of Dental Sciences (ABSMIDS), Department of Conservative Dentistry and Endodontics, Deralakatte, Mangaluru, India

**Keywords:** dental pulp, dentine–pulp complex, heat-shock response, intrapulpal temperature, minimally invasive dentistry, scoping review, thermal conductivity, thermal micro-insults

## Abstract

**Background:**

Thermal changes generated during routine dental procedures have traditionally been evaluated only in relation to acute pulpal injury and irreversible temperature thresholds. A growing body of experimental evidence suggests that repeated low-grade heat exposure may produce cumulative sub-clinical biological changes within the dentine–pulp complex, a phenomenon we here group under the working term “thermal micro-insults” (TMI).

**Objective:**

To map the available literature on sub-threshold, sub-lethal and sub-ablative thermal exposure of the dentine–pulp complex during routine restorative and endodontic procedures, and to identify concepts, mechanisms, evidence gaps and clinically relevant thermal parameters.

**Methods:**

A scoping review was conducted following the Joanna Briggs Institute (JBI) methodology and reported according to the PRISMA Extension for Scoping Reviews (PRISMA-ScR). PubMed/MEDLINE, Scopus, Web of Science and the Cochrane Central Register of Controlled Trials were searched from January 1965 to May 2026 using combined keywords covering thermal injury, intrapulpal temperature, dentine–pulp complex, heat-shock response and the specific dental procedures under consideration. *In vitro*, *ex vivo*, *in vivo* animal and human studies, and prior narrative or systematic reviews were eligible. Two reviewers independently screened records; disagreements were resolved by discussion.

**Results and discussion:**

Heat generated during cavity preparation, finishing/polishing, photopolymerisation, ultrasonic instrumentation and laser use can transiently raise intrapulpal temperature. Even when the classical 5.5 °C Zach and Cohen threshold is not exceeded, repeated sub-threshold exposures have been associated in *in vitro* and animal studies with heat-shock-protein expression (HSP25, HSP27, HSP70), altered mitochondrial function and reactive-oxygen-species generation, up-regulation of pro-inflammatory cytokines (IL-1β, IL-6, IL-8, TNF-α), activation of thermosensitive TRPV1/TRPA1 channels, and long-term changes such as reparative dentine deposition, focal fibrosis and calcification. Current evidence is dominated by *in vitro* work using extracted teeth without pulpal microcirculation; clinical validation and standardised sub-threshold thermal-dose descriptors are lacking.

**Conclusions:**

TMI should be viewed as a plausible but still developing hypothesis rather than an established clinical entity. Recognising cumulative low-grade heat exposure as a potentially modifiable biological factor may complement existing minimally invasive restorative and endodontic principles, guide the design of low-heat instrumentation and cooling strategies, and inform future *in vivo* research.

## 1 Introduction

The tooth is a thermally heterogeneous organ. Its thermophysical behaviour is governed by the low thermal conductivity and diffusivity of enamel and dentine, and by the vascularised, heavily innervated pulp, which contributes to heat dissipation through convective blood flow and to thermal sensation through primary afferent fibres (1). Under physiological conditions, human teeth are continuously exposed to thermal fluctuations from food, beverages and ambient air, which are transferred through enamel and dentine to the pulp (1, 2).

In addition to physiological thermal loading, teeth are subjected to iatrogenic heat generation during virtually every restorative and endodontic procedure. Cavity preparation, finishing and polishing, photopolymerisation of resin-based composites, ultrasonic instrumentation and laser irradiation all convert mechanical, chemical or optical energy into localised thermal energy that is conducted toward the pulp (3). Direct *in vivo* measurement of intrapulpal temperature in humans is not clinically feasible; consequently, the literature relies heavily on *in vitro* models, animal studies and mathematical simulations, which have generated widely divergent estimates of pulpal heat load (2).

Classical dental thermal-injury research has focused on identifying discrete temperature thresholds above which irreversible pulpal damage occurs, most notably the 5.5 °C intrapulpal rise associated by Zach and Cohen with irreversible pulpitis in approximately 15% of exposed teeth (4). This threshold framework has been extremely useful in defining upper safety limits but says little about the biological fate of the pulp when temperatures remain below the injury threshold, particularly when such exposures are repeated across a single visit or across successive appointments. Recent experimental data on heat-shock protein expression, mitochondrial dysfunction, oxidative stress and cytokine release in pulp cells exposed to fever-range or sub-lethal heat suggest that biologically meaningful changes may occur well below the classical thresholds (3, 5–9).

For the purposes of this review we use the working term “thermal micro-insults” (TMI) to describe cumulative sub-ablative, sub-lethal and sub-symptomatic thermal exposures of the dentine–pulp complex delivered during routine dental procedures. The term is not intended as a new clinical diagnosis, but as an organising concept that unifies related descriptors already present in the literature (sub-threshold thermal injury, mild hyperthermia, cumulative thermal stress, biologic heat stress; see Section 3.2).

The objectives of the present scoping review are: (i) to map the range and type of evidence on sub-threshold thermal exposure of the dentine–pulp complex during routine dental procedures; (ii) to summarise the reported biological responses of the pulp to such exposures; (iii) to identify procedural sources and modifiers of pulpal heat load; and (iv) to highlight methodological limitations and gaps that should be addressed by future *in vivo* and clinical work.

## 2 Materials and methods

### 2.1 Study design and reporting

This work was designed as a scoping review, following the framework of Arksey and O'Malley, later refined by Levac et al. and the Joanna Briggs Institute (JBI) methodology for scoping reviews (10). Reporting followed the Preferred Reporting Items for Systematic Reviews and Meta-Analyses Extension for Scoping Reviews (PRISMA-ScR) checklist (11).

### 2.2 Review question

The primary review question was formulated using the PCC (Population, Concept, Context) framework: In the human dentine–pulp complex (Population), what is known about sub-threshold, sub-lethal and cumulative thermal exposure (“thermal micro-insults”, Concept) generated during routine restorative and endodontic procedures (Context)?

### 2.3 Eligibility criteria

**Inclusion criteria:**

- Peer-reviewed original research (*in vitro*, *ex vivo*, *in vivo* animal or human) or reviews addressing intrapulpal or intradentinal temperature changes during dental procedures.
- Studies reporting biological, molecular, cellular or histological responses of pulp or dentine cells to heat exposure below or around the classical 5.5 °C threshold.
- Studies on procedural variables affecting pulpal heat load (bur design, handpiece type, coolant flow, curing-light output, ultrasonic power, laser parameters, polishing systems).
- English-language publications from January 1965 (Zach and Cohen) to May 2026.

**Exclusion criteria:**

- Editorials, letters, conference abstracts without full data, and single case reports.
- Studies on cryotherapy or deliberately therapeutic hyperthermia unrelated to routine dental procedures.
- Publications for which the full text could not be retrieved.

### 2.4 Information sources and search strategy

Four electronic databases were searched: PubMed/MEDLINE, Scopus, Web of Science Core Collection, and the Cochrane Central Register of Controlled Trials. The reference lists of included articles and of key narrative reviews (Lin et al. 2010; Lau et al. 2023) were hand-searched. Grey literature was identified through Google Scholar (first 200 records) and ProQuest Dissertations and Theses.

The following search string was adapted to each database syntax: (“intrapulpal temperature” OR “pulp chamber temperature” OR “pulp heating” OR “thermal injury” OR “thermal micro-insult*” OR “heat shock” OR “hyperthermia”) AND (“dental pulp” OR “dentin*” OR “dentine” OR “odontoblast*”) AND (“cavity preparation” OR “bur” OR “handpiece” OR “polishing” OR “finishing” OR “light curing” OR “photopolymerisation” OR “ultrasonic” OR “scaler” OR “laser” OR “Er:YAG” OR “Er,Cr:YSGG”). The final search was executed on 15 May 2026.

### 2.5 Study selection

Records were exported to Rayyan for de-duplication. Two reviewers independently screened titles and abstracts against the eligibility criteria; potentially relevant full texts were retrieved and assessed. Disagreements were resolved through discussion, and a third reviewer was consulted where consensus could not be reached. Because this is a scoping review, no formal risk-of-bias appraisal was performed; however, methodological features (*in vitro* vs. *in vivo*, presence or absence of pulpal microcirculation, thermocouple placement, sample size) were extracted to inform the narrative critical appraisal in Section 6.

### 2.6 Data extraction and synthesis

A standardised data-charting form captured: author, year, country; study design and model; procedure and instrumentation studied; thermal parameters (peak *Δ*T, exposure duration, coolant flow); biological or histological endpoints; and main findings. Data were synthesised narratively and grouped by (a) procedural source of thermal load and (b) biological level of response (molecular, cellular, tissue-level, clinical).

### 2.7 Search results

The initial search retrieved 612 records from the four databases plus 24 additional records from hand-search and grey literature (*n* = 636). After removal of 178 duplicates, 458 unique records were screened by title and abstract; 338 were excluded at this stage. Of the 120 full-text articles assessed for eligibility, 74 were excluded (see Figure 1). Forty-six studies met all inclusion criteria and were included in the final qualitative synthesis. A PRISMA-ScR flow diagram is provided as Figure 1. Because of the heterogeneity of designs and outcome measures, no quantitative pooling was attempted.

**Figure 1:**
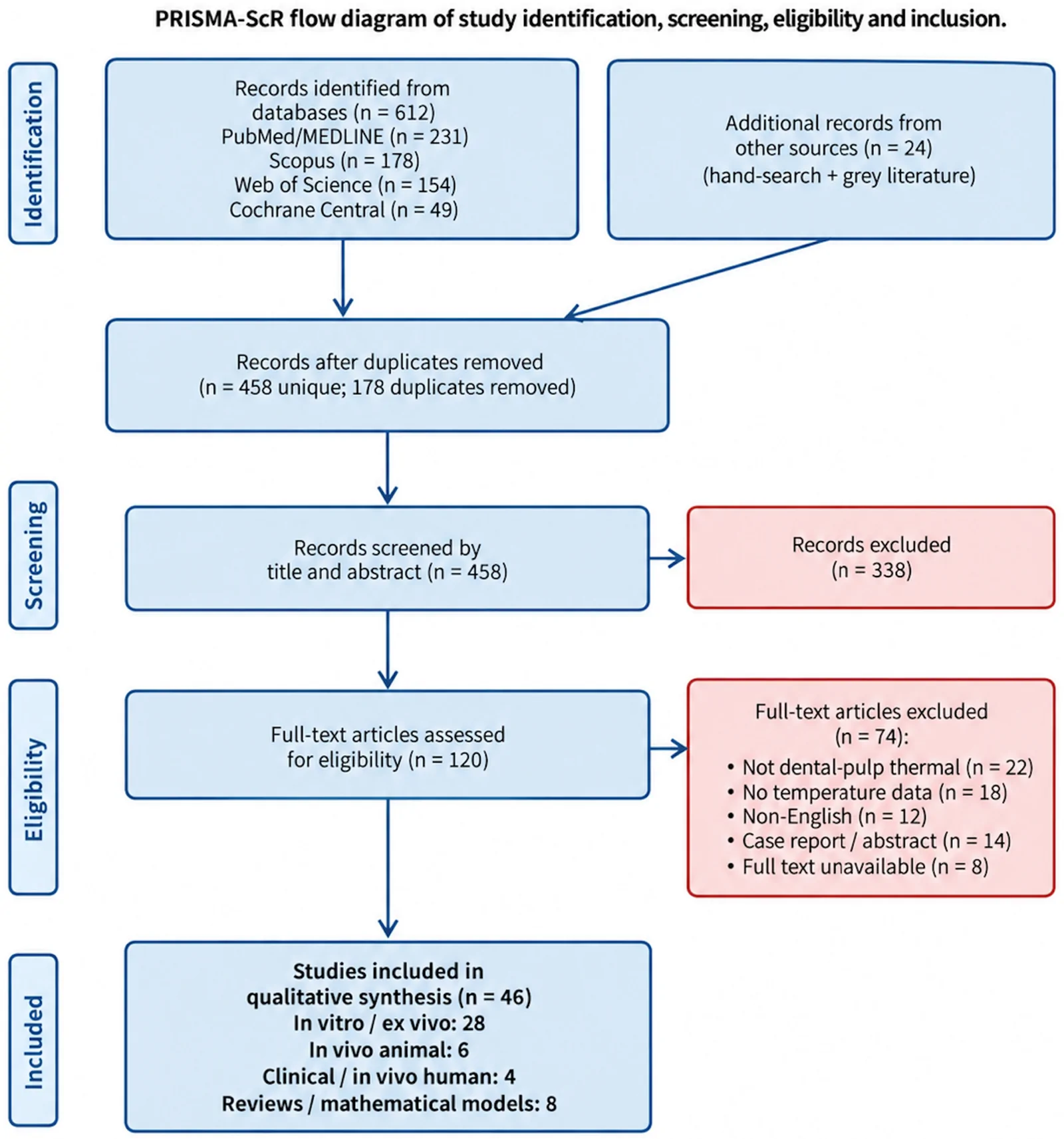
PRISMA-ScR flow diagram of study identification, screening, eligibility and inclusion. Legend: Flow of records through the four PRISMA-ScR phases (Identification, Screening, Eligibility and Inclusion). Boxes in blue indicate the number of records at each stage; red boxes indicate the number of records excluded, with reasons for full-text exclusion. Databases searched were PubMed/MEDLINE, Scopus, Web of Science Core Collection and the Cochrane Central Register of Controlled Trials; additional records were identified through hand-search of reference lists of key reviews and grey literature (Google Scholar, ProQuest Dissertations and Theses). Diagram prepared following the PRISMA-ScR reporting checklist of Tricco et al. (11).

## 3 Historical evolution and terminology

### 3.1 Historical overview

Infection management and prevention of macroscopic tissue damage have historically dominated the endodontic literature, because microbial contamination is the principal cause of pulpal and periapical disease (5). Early thermal-injury studies focused almost exclusively on identifying temperature thresholds above which permanent damage, such as pulpal necrosis and bone resorption, could be demonstrated histologically (3, 4). Zach and Cohen's seminal 1965 rhesus-monkey study established 5.5 °C as the intrapulpal rise associated with irreversible pulpitis in approximately 15% of teeth (4); subsequent *in vitro* and *in vivo* work refined this threshold and defined safety recommendations for a range of instruments (Table 1) (2, 3, 12–15).

**Table 1:** Historical evolution of thermal-injury research in dentistry: key findings, safety thresholds and temperature-monitoring methods.

Year	Author (Ref.)	Study type	Key finding	Reported threshold/temperature	Monitoring method
1965	Zach and Cohen (4)	*In vivo* (monkey)	5.5 °C intrapulpal rise → irreversible pulpitis in ∼15% of teeth	5.5 °C (“classical” upper safety limit)	Thermocouple in pulp chamber
1970	Brown et al. (3)	*In vitro* (extracted)	Defined thermal conductivity/diffusivity of enamel and dentine	Enamel k ≈ 0.9 W m⁻^1^ K⁻^1^; dentine k ≈ 0.6	Thermocouple + heat-flow apparatus
1985	Pashley (12)	Review	Dentinal fluid movement modulates heat transfer through tubules	N/A (mechanistic)	Permeability model
1989	Hibst and Keller (13)	*In vitro* (laser)	Er:YAG laser ablates dental hard tissue with low residual heat	*Δ*T < 2 °C with water spray	Thermocouple + IR
1990	Saunders (14)	*In vivo* (animal; ferret canine)	Thermomechanical compaction of gutta-percha produced measurable temperature elevations on the external root surface; temperature elevations dissipated more rapidly in vivo than in vitro	External root-surface temperature elevation during thermomechanical compaction; the study evaluated whether rises approaching or exceeding 10 °C could occur under in vivo conditions	Temperature measurement at the mid-root external surface during thermomechanical compaction
1994	Andersen et al. (8)	*In vivo* (human)	Moderate cooling and warming altered pulpal blood flow, with substantial inter-individual variability	Cooling from 33 °C to 5 °C reduced pulpal blood flow to approximately 80% of control on average; warming to 39 °C produced a small reduction in most subjects	Laser Doppler flowmetry.
1997	Baldissara et al. (2)	*In vitro*/clinical	Injury is time-temperature dependent, not a single threshold	≥ 5.5 °C at ≥ 30 s exposure	Fine thermocouple
1998	Nicoll and Peters (16)	*In vitro* (ultrasonic)	Dentine *Δ*T up to 11 °C during ultrasonic scaling without irrigation	> 5.5 °C at low irrigation	Thermocouple on dentine
2004	Ozturk et al. (1)	*In vitro* (cavity prep)	Intrapulpal *Δ*T strongly modulated by remaining dentine thickness (RDT)	*Δ*T reduced∼60% when RDT ≥ 2 mm	Thermistor in pulp chamber
2008, 2019	Jakubinek, Vinagre et al. (17, 18)	*In vitro* (LED curing)	High-power LEDs raise pulp-dentine *Δ*T 3–8 °C, > 10 °C with thin RDT	3–8 °C (thin cavities > 10 °C)	Thermocouple + IR
2010	Lin et al. (19)	Review/modelling	Mathematical models of pulp heat transfer including microcirculation	Blood flow lowers peak *Δ*T∼25%–40%	FEM/bio-heat equation
2014	Morotomi et al. (20)	*In vitro* (cell)	Fever-range heat induces HSP70 and thermotolerance in odontoblasts	Adaptive at 39–41 °C	Cell viability + immunoblot
2023	Lau et al. (15)	Review	Cumulative sub-threshold heat may contribute to long-term pulpal decline	Reinforces 5.5 °C only as reference	IR thermography and thermocouple
2024	Liu et al. (21)	*In vitro* (MDPC-23)	Mild heat stress promotes odontoblast differentiation via YAP	Adaptive at 40 °C, 30 min	Cell assays

Chronological summary of milestone studies shaping the current understanding of thermal impact on the dental pulp. “Reported threshold/temperature” indicates the principal quantitative outcome as originally reported; values should be interpreted in the context of each study's design (*in vitro* versus *in vivo*, presence or absence of pulpal microcirculation, thermocouple placement). HSP70 = heat-shock protein 70; RDT = remaining dentine thickness; FEM = finite-element modelling; IR = infrared.

Technical limitations of the era – bulky thermocouples, absence of real-time infrared imaging, and difficulties in reproducing pulpal microcirculation – constrained the interpretation of these early experiments. As instrumentation has evolved (fine-tip thermocouples, thermistors, IR thermography, computational modelling), interest has shifted from single-shot threshold events toward the biology of moderate, repeated, sub-threshold exposures (3, 15).

### 3.2 Terminology and positioning of “thermal micro-insults”

Several overlapping terms already exist in the literature: sub-threshold thermal injury, mild hyperthermia, cumulative thermal stress and biologic heat stress. These are not synonymous. Sub-threshold thermal injury usually denotes a single exposure below a defined injury threshold; mild hyperthermia is a broader biophysical descriptor (typically 39–42 °C tissue temperature) used across medicine, including oncology; cumulative thermal stress refers primarily to repeated exposures without necessarily specifying magnitude; and biologic heat stress describes the resulting cellular response rather than the stimulus itself.

In this review “thermal micro-insults” (TMI) is used as an umbrella working term describing: (i) exposures that are sub-ablative (below the temperature at which structural ablation, vaporisation or carbonisation of dentine or pulp occurs); (ii) sub-lethal at the cellular level; (iii) sub-symptomatic clinically (i.e., not producing immediate pain, sensitivity or radiographic change); and (iv) cumulative in nature, occurring repeatedly during and between routine dental procedures. This framing does not replace the existing terminology; rather, it groups it around the specific dental context in which each exposure is small, largely unnoticed, but potentially additive over the biological life of a tooth. The added scientific value of the term is thus primarily heuristic – it directs attention to (a) sub-threshold events during routine dentistry that are rarely reported and (b) their cumulative impact – rather than describing a novel biological mechanism.

## 4 Structure and thermal properties of the tooth

The tooth acts as a graded thermal barrier. Enamel and dentine have low thermal conductivity (≈ 0.9 W m⁻^1^ K⁻^1^ and 0.6 W m⁻^1^ K⁻^1^ respectively) and low thermal diffusivity, which delay the transmission of surface temperature changes to the pulp (1, 19). The coefficient of thermal expansion (CTE) of enamel is approximately 11.4 × 10⁻⁶/ °C and that of dentine 8.3 × 10⁻⁶/ °C; the relatively low CTE values contribute to the dimensional stability of dental tissues under repeated thermal cycling (19).

Heat dissipation in dentine is modulated by dentinal tubule density and orientation; outward and inward fluid movement within the tubules can act as either a heat sink or a facilitator of heat transfer depending on flow direction and rate (12, 19). Pulpal blood flow provides an additional, highly efficient convective mechanism for heat dissipation, which is absent in extracted-tooth models and represents one of the principal limitations of *in vitro* thermal studies (19, 22). Remaining dentine thickness (RDT) is the single most important structural determinant of intrapulpal temperature rise reported in the literature; RDT ≤ 0.5 mm has repeatedly been associated with a disproportionately large pulpal temperature response, whereas RDT ≥ 2 mm confers substantial protection under most experimental conditions (15, 23).

## 5 Dentistry as a thermal discipline

Endodontics and conservative dentistry are fundamentally based on the transfer of thermal, mechanical, chemical and optical energy into the biological substrate of the tooth. Thermal energy is produced during cavity preparation (friction between rotary instruments and dentine), during finishing and polishing (frictional heat and abrasive interaction), during photopolymerisation (light-source irradiance and the exothermic reaction of the resin), during ultrasonic instrumentation (acoustic microstreaming, cavitation and stack heating), during laser irradiation (absorption of coherent light by dental hard tissue), and during thermoplastic obturation (direct conductive heating from carriers or heated pluggers) (3, 23–26).

Despite the ubiquity of these thermal loads, routine clinical practice contains no established mechanism for monitoring intrapulpal temperature. Safety in conservative dentistry is defined almost exclusively by rapid macroscopic outcomes – absence of pulpal exposure, favourable radiographic follow-up and short-term postoperative comfort – with limited attention paid to sub-clinical biological consequences of thermal load.

### 5.1 The 5.5 °C threshold revisited

The 5.5 °C intrapulpal rise reported by Zach and Cohen (4) is often quoted as an absolute upper safety limit. This interpretation should be qualified. First, the study was performed in rhesus monkey teeth with externally applied heat, and the histological endpoint was defined at 8 weeks. Second, subsequent work has consistently shown that pulpal responses to thermal stress are multifactorial, depending on the magnitude of temperature rise, the duration of exposure, remaining dentine thickness, pulpal blood flow and inter-individual biological variability (2, 27). Thermal injury is therefore increasingly recognised as a time–temperature-dependent phenomenon rather than one governed by a single temperature threshold.

In the present review the 5.5 °C value is used only as a historical reference point, and the underlying evidence base is treated with appropriate caution. Most temperature/recovery-time data originate from *in vitro* studies on extracted teeth, which lack pulpal microcirculation and can therefore be expected to overestimate the peak temperature attained during a clinical procedure of the same nominal parameters. Any thresholds and “safe” exposure times cited in the literature should thus be interpreted as conservative laboratory-based estimates rather than definitive clinical guidelines (19, 22).

## 6 Thermal micro-insults: working definition and underlying concepts

As defined above, TMI describes low-grade thermal exposures of the dentine–pulp complex that do not individually produce necrosis, ablation or overt clinical symptoms, but that may aggregate over time to influence pulpal biology. Three qualifiers are essential:

- Sub-ablative – the temperature remains below the level required for vaporisation or carbonisation of dentine or pulp; overall tissue architecture is preserved.
- Sub-lethal – cellular damage is reversible or only partially reversible; protein denaturation and enzymatic disturbance may occur but do not cause immediate cell death.
- Sub-symptomatic – no immediate pain, sensitivity or radiographic change is produced, so the exposure is easily overlooked in routine practice.

Precise temperature and exposure-time boundaries for TMI in the dentine–pulp complex have not been experimentally defined. The concept therefore currently rests on biological responses observed below recognised injury thresholds rather than on quantitatively agreed thermal limits. This is a critical evidence gap and one of the primary drivers for future *in vivo* studies (see Section 10).

## 7 Sources of thermal micro-insults in routine dentistry

### 7.1 High-speed handpieces, burs and cavity preparation

High-speed dental handpieces (HSDHs) – either air-turbine or electric micromotor-driven – generate heat by friction between the rotating bur and dentine. Reported intrapulpal temperature rises during cavity preparation without adequate coolant range from 3 °C to more than 15 °C depending on load, bur wear, RDT and coolant flow (15, 23, 24, 28). Electric handpieces have been reported to generate lower operating-head temperatures than air-turbines under load, primarily because of more efficient torque delivery and concentric bur rotation (29). Bur design is a critical but often under-emphasised variable: worn burs, aggressive diamond grits and larger head diameters all increase frictional heat, while shorter cutting bursts, lighter load and use of new burs reduce it (23, 30). Diamond burs typically produce higher intrapulpal temperatures than tungsten-carbide burs at equivalent parameters, and their thermal behaviour deteriorates rapidly with wear.

Coolant delivery is decisive. Air coolant alone is inadequate to prevent pulpal heat build-up; air–water spray is required, and international standards (ISO 14457 and related guidance) recommend a water flow rate of ≥ 30 mL min⁻^1^, with 50 mL min⁻^1^ frequently used as a practical upper limit that also preserves visibility (24). Below this flow rate, thermal-injury risk rises sharply, particularly when RDT falls below 1 mm.

### 7.2 Finishing, polishing and adjustment of restorations and ceramics

Finishing and polishing procedures are frequently perceived as “finishing touches” with limited biological consequence, but multiple *in vitro* studies demonstrate clinically significant temperature rises when polishing is performed dry or at high rotational speed (31). Dry adjustment or high-speed polishing of yttria-stabilised zirconia crowns has been reported to elevate the surface temperature by more than 30 °C and to raise intrapulpal temperature well beyond the conventional 5.5 °C threshold; polishing at 20 000 rpm without water spray consistently exceeds this limit (32, 33). Recommended mitigations include using continuous water irrigation, limiting rotational speed (< 10 000 rpm for high-gloss polishing), applying light pressure and using intermittent contact.

### 7.3 Photopolymerisation and light curing

Light curing produces heat by two mechanisms operating in parallel: absorption of radiant energy from the curing unit and the exothermic polymerisation reaction of the resin. Modern high-output LED curing units can produce peak pulpal temperature increases of 3–8 °C *in vitro*, with certain high-power devices and bulk-fill composites exceeding 10 °C when RDT is minimal (17, 18, 34–36). Temperature rise is influenced by curing time, irradiance, tip-to-composite distance, composite type and volume, and use of soft-start protocols. In deep cavities with minimal RDT the cumulative effect of multiple curing cycles per restoration is of particular relevance to the TMI concept.

### 7.4 Ultrasonic instrumentation and scalers

Ultrasonic tips and endodontic ultrasonic instruments generate heat through direct friction between the vibrating tip and the tooth surface and through acoustic microstreaming and cavitation within irrigants (16, 37, 38). Nicoll and Peters demonstrated that dentine surface temperature can rise by 4–11 °C during periodontal ultrasonic scaling depending on power setting and irrigation flow. Ultrasonic activation of endodontic irrigants and ultrasonic post removal have been associated with external root-surface temperature increases that occasionally exceed 10 °C when coolant is inadequate (37). Continuous irrigation (typically ≥ 20–30 mL min⁻^1^ during scaling), moderate power settings and intermittent tip contact are the principal thermal-control strategies.

### 7.5 Lasers and thermoplastic obturation

Erbium-family lasers (Er:YAG at 2 940 nm and Er,Cr:YSGG at 2 780 nm) offer high ablation efficiency in dental hard tissue with relatively low thermal side effects when used with water spray, and several *in vitro* studies report intrapulpal temperature rises below 2 °C for cavity preparation and caries removal (13, 39–41). Nd:YAG and diode lasers, in contrast, generate higher thermal loads on the pulpal axial wall and require careful parameter control. Thermoplastic gutta-percha obturation techniques transmit heat to the root surface; peak external root temperatures usually remain below 10 °C above baseline when standard operator technique is used, but may exceed this in thin-walled roots.

## 8 Biological response of the dentine–pulp complex to thermal micro-insults

The dentine–pulp complex responds to sub-threshold thermal exposure through a coordinated hierarchy of molecular, cellular, tissue-level and neural events. The following sub-sections summarise the evidence for each level, while highlighting the predominance of *in vitro* data and the associated methodological caveats.

### 8.1 Molecular response: heat-shock proteins and stress signalling

Heat-shock proteins (HSPs) act as molecular chaperones that stabilise unfolded proteins and inhibit apoptosis. In dental pulp, HSP25, HSP27 and HSP70 have all been documented in response to thermal, mechanical and chemical stress (42–44). Chen et al. demonstrated increased HSP70 immunolocalisation during reparative dentine formation after cavity preparation (43). Morotomi et al. showed that continuous fever-range heat stress (39–41 °C) induced HSP70 expression and thermotolerance in odontoblast-lineage cells without loss of viability (20). Recent work by Liu et al. reported that mild heat stress enhanced odontoblast differentiation in MDPC-23 cells via a YAP-dependent pathway, indicating that low-grade heat can have context-dependent adaptive as well as injurious effects (21). HSP up-regulation is therefore a sensitive indicator of exposure to sub-lethal heat and provides a biological bridge between conventional threshold thinking and the TMI concept.

### 8.2 Cellular response: mitochondrial function, oxidative stress and cytokines

Sub-lethal thermal exposure impairs mitochondrial ATP production and increases the generation of reactive oxygen species (ROS), producing an oxidative imbalance that can persist after the stimulus has been removed (45, 46). Chang et al. showed that heat exposure at 42 °C for 30 min activated the antioxidant system and up-regulated interleukin-8 (IL-8) and its receptor in human dental pulp cells (45). Additional evidence from pulp-inflammation studies documents increased expression of IL-1β, IL-6 and TNF-α in response to combined thermal and chemical stress (47). The magnitude of the response depends on temperature, duration of exposure and cell type, and appears to be cumulative when repeated exposures are delivered (48).

### 8.3 Tissue-level response: microcirculation, fibrosis and tertiary dentine

*In vivo* studies demonstrate that thermal stimuli can alter pulpal blood flow, with responses varying according to the direction and magnitude of the thermal stimulus and between individuals (8). Chronic sub-threshold thermal exposure has been associated with tertiary (reactionary or reparative) dentine deposition, focal pulpal fibrosis and dystrophic calcification, changes that are often asymptomatic and only detected on histological examination of extracted teeth (9, 22). Absence of symptoms therefore does not equate to biological quiescence.

### 8.4 Neural response: thermosensitive TRP channels and sensitisation

The dental pulp is densely innervated by A-*δ* and C fibres expressing thermosensitive transient receptor potential (TRP) channels, notably TRPV1 (heat), TRPA1 (cold and irritants), TRPV2 (noxious heat) and TRPC5 (cold) (7, 49–51). Odontoblasts themselves express functional TRPV1, TRPA1 and TRPV4 channels, supporting a peripheral sensory role for these cells (7, 50). Repeated activation of TRPV1 by mild heat can lead to peripheral and central sensitisation, potentially contributing to chronic post-restorative sensitivity that clinicians frequently describe but rarely quantify. Importantly, TRPV1-expressing pulpal neurons appear to play a more prominent role after injury than in healthy pulp, supporting the idea that the TMI concept is more clinically relevant in already-stressed teeth (deep caries, previously restored teeth) (49).

### 8.5 Integrating levels: the pulp as an adaptive system

Molecular chaperoning (HSPs), redox buffering, cytokine-mediated signalling, vascular regulation and neural gating together allow the pulp to withstand transient thermal challenges. When exposures remain modest, adaptation dominates; with repetition or when RDT is minimal, adaptive capacity may be exceeded, producing histologically detectable but clinically silent changes. This biological continuum – rather than a single injury threshold – is at the heart of the TMI concept. Figure 2 provides an integrated graphical summary of the cascade from odontoblastic response through pulpal inflammation and dentine changes to potential clinical outcomes.

**Figure 2:**
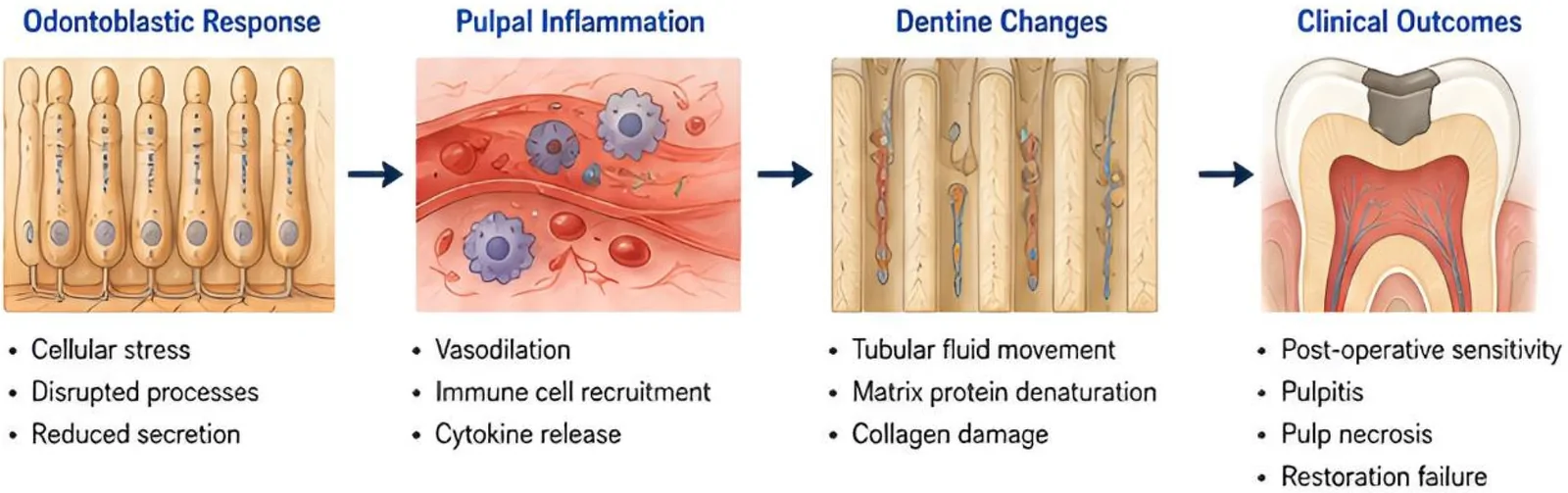
Graphical summary of the biological cascade triggered by thermal micro-insults in the dentine–pulp complex. Sequential representation of the four hierarchical levels of pulpal response to sub-threshold heat. (1) Odontoblastic response — cellular stress, disruption of odontoblast processes and reduced secretory activity; (2) Pulpal inflammation — vasodilation, immune-cell recruitment and release of pro-inflammatory cytokines (IL-1β, IL-6, IL-8, TNF-α); (3) Dentine changes — outward or inward tubular fluid movement, matrix protein denaturation and collagen damage; (4) Clinical outcomes — postoperative sensitivity, reversible or irreversible pulpitis, pulp necrosis and restoration failure. The figure illustrates that a single TMI rarely produces overt clinical signs, whereas cumulative or repeated exposure can drive silent progression toward each successive stage.

## 9 Clinical implications and evidence-informed practical considerations

Although the TMI hypothesis is still developing, the available literature identifies several practical measures that may reduce procedural thermal load. These are consolidated in Table 2 and visually summarised in Figure 3. Because the supporting evidence is derived predominantly from *in vitro* and animal studies, these measures should be interpreted as evidence-informed practical considerations rather than formal consensus recommendations or validated clinical guidelines. Quantitative values are retained where they provide useful experimental or equipment-related reference points, but they should not be construed as clinically validated biological safety thresholds.

**Table 2:** Evidence-informed practical considerations for controlling thermal load during common dental procedures.

Procedure	Key parameter	Evidence-informed practical consideration	Supporting refs
Cavity preparation (rotary)	Coolant delivery	Use air-water spray with correct port alignment. Experimental handpiece studies and equipment-related guidance commonly use flow rates ≥ 30 mL min⁻^1^, with∼50 mL min⁻^1^ in some protocols; these values are reference parameters rather than validated pulpal safety thresholds.	[(24), ISO 14457]
Cavity preparation (rotary)	Remaining dentine thickness	Preserve as much RDT as clinically feasible and use intermittent cutting in deep cavities. Experimental models indicate lower thermal transmission with RDT around ≥ 1–2 mm; these values are not validated clinical cut-offs.	(1, 15, 23)
Cavity preparation (rotary)	Bur/handpiece	Replace worn burs and use sharp/new instruments; tungsten-carbide burs or electric handpieces may be considered where appropriate to reduce frictional heat.	(23, 29, 30)
Zirconia/ceramic adjustment	Rotational speed	Use dedicated polishing systems with continuous water and avoid dry adjustment. Laboratory studies support lower-speed polishing; values around ≤ 10 000 rpm have been evaluated experimentally but should not be regarded as a universal clinical limit.	(31–33)
Photopolymerisation	Curing protocol	Follow manufacturer-recommended exposure times and maintain appropriate tip position; moderate irradiance and soft-start/pulsed modes may be considered in deep preparations. RDT < 1 mm represents an experimental higher-risk condition rather than a validated clinical cut-off.	(17, 18, 34–36)
Ultrasonic scaling	Irrigation/power	Use continuous coolant, moderate power and intermittent contact, and avoid prolonged single-site instrumentation. Coolant flow rates of approximately 20–30 mL min⁻^1^ have been evaluated experimentally but are not validated clinical thresholds.	(16, 37, 38)
Endodontic ultrasonic activation	Power/duration	Use short activation cycles with an adequate irrigant reservoir, particularly in thin-walled roots. Durations of ≤ 20 s have been evaluated experimentally and should not be interpreted as a universal safety limit.	(16, 37)
Laser use (hard tissue)	Wavelength/cooling	Use water spray with Er:YAG or Er,Cr:YSGG systems and follow device/manufacturer specifications for pulse energy and repetition rate; thermal effects are parameter- and device-dependent.	(13, 39–41)
Thermoplastic obturation	Carrier/plugger temperature	Follow manufacturer-recommended technique and exercise additional caution in thin-walled roots and closed apices; the available evidence does not establish a single validated temperature threshold for clinical use.	(14, 15)
Session planning	Cumulative thermal load	Where multiple heat-generating procedures are required on the same tooth, consider limiting unnecessary cumulative exposure and allowing cooling intervals. No validated cumulative thermal-dose or RDT-adjusted exposure metric is currently available.	[(15), this review]

Practical considerations derived from the studies identified in this scoping review. They are not formal consensus recommendations or validated clinical guidelines. Most quantitative values originate from *in vitro* or animal studies, while some device-related parameters reflect equipment standards or manufacturer guidance. Accordingly, numerical values should be interpreted as experimental or operational reference parameters rather than biological safety cut-offs. RDT = remaining dentine thickness; TC = tungsten carbide; ISO 14457 = International Standard for dental handpieces.

**Figure 3:**
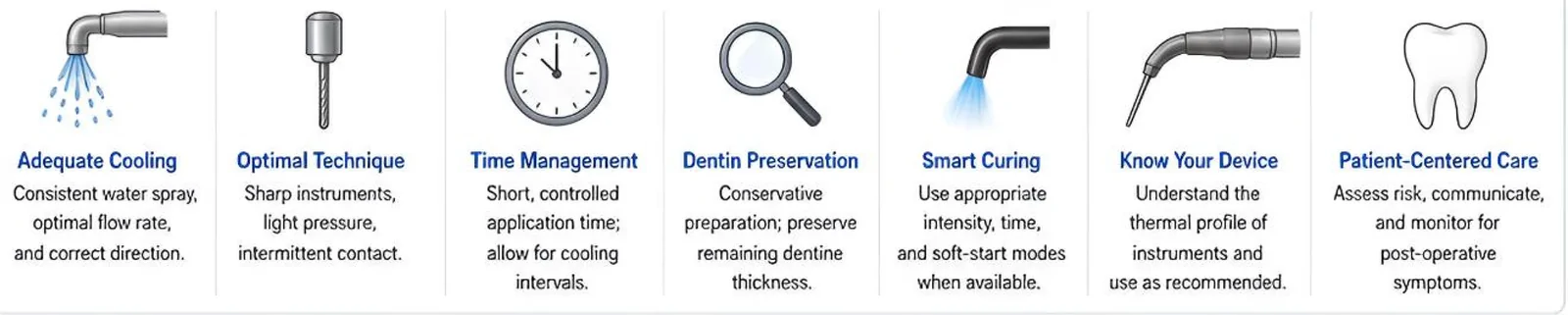
Practical seven-pillar clinical framework for minimising thermal micro-insults during routine dental procedu**res.** (1) Adequate cooling - consistent water spray, adequate flow and correct port direction; experimentally studied or equipment-related values such as ≥ 30 mL min⁻^1^ should not be interpreted as validated pulpal safety thresholds; (2) Optimal technique - sharp instruments, light pressure and intermittent contact; (3) Time management - short, controlled application times with brief cooling intervals; (4) Dentine preservation - conservative cavity preparation and preservation of remaining dentine thickness; (5) Smart curing - appropriate irradiance, curing time and soft-start modes; (6) Know your device - awareness of the thermal profile of each instrument used and adherence to manufacturer recommendations; (7) Patient-centred care - risk assessment, communication and structured monitoring for postoperative symptoms. The seven pillars summarise the evidence-informed practical considerations in Table 2 and are intended as precautionary principles pending clinical validation.

## 10 Limitations of the current evidence base

Several limitations must be acknowledged when interpreting the TMI literature:

- Predominance of *in vitro* evidence: most temperature measurements are obtained from extracted teeth, which lack pulpal microcirculation and therefore likely overestimate peak intrapulpal temperature (19, 22).
- Lack of standardised thermal thresholds for sub-threshold, sub-lethal and sub-ablative exposure: existing thresholds are largely inherited from single-event studies and have not been formally validated for cumulative low-grade heat.
- Limited clinical validation: with the exception of Zach and Cohen's animal work (4) and a small number of *in vivo* studies (8, 14), virtually no data directly link routine sub-threshold thermal exposures to long-term human pulpal outcomes.
- Influence of pulpal blood flow: convective heat loss through pulpal blood flow is a major, difficult-to-model variable; individual variation in pulp vascularity has not been systematically characterised.
- Interpatient biological variability: age-related dentine sclerosis, dentinal-tubule density, pulp volume and prior restorative history all modify thermal response but are rarely controlled in existing studies (15, 28).
- Absence of longitudinal human studies: no prospective clinical trial to date has tracked pulpal health as a function of documented thermal exposure across the working life of a tooth.
- Heterogeneity in study design: measurement technique (thermocouple placement, thermistor, IR thermography), cavity dimensions, coolant delivery and load control differ widely between studies, limiting quantitative synthesis.
- Publication bias: negative findings (e.g., procedures that do not produce measurable pulpal temperature rise) are under-reported.

• Because of these limitations, the conclusions drawn in this review are deliberately framed as hypothesis-generating rather than definitive.

## 11 Future perspectives and emerging technologies

Progress in this field will require both methodological refinement and technological innovation. Key priorities include:

- Real-time intrapulpal temperature monitoring using MEMS-based microsensors, fibre-optic temperature probes and non-contact infrared thermography systems adapted to the intraoral environment (52).
- Smart handpieces with feedback-controlled cooling and integrated temperature sensors, capable of automatically modulating rotational speed and coolant flow in response to real-time pulpal temperature estimates.
- Improved cooling systems, including cryogenic gas assistance, coolant temperature control and micro-jet water delivery.
- Advanced curing units with predictive thermal-limitation algorithms, soft-start and pulsed curing modes, and integrated pulp-temperature estimation.
- Biomarker-based assessment of pulpal thermal stress *in vivo*, including HSP70, IL-8 and mitochondrial-dysfunction markers in gingival crevicular fluid or dentinal fluid, to correlate thermal exposure with sub-clinical pulp response.
- *ex vivo* perfused-tooth models that replicate pulpal microcirculation to bridge the current gap between *in vitro* and clinical studies.
- Machine-learning models that integrate patient-specific factors (age, RDT, restorative history, pulp volume) with procedural parameters to predict individual thermal risk.
- Prospective clinical trials linking documented cumulative thermal exposure with long-term pulpal outcomes, including postoperative pain, pulp vitality and pulp calcification.

## 12 Conclusion

Available evidence, drawn predominantly from *in vitro* and mechanistic studies, indicates that routine dental procedures can generate sub-threshold thermal loads that are individually modest but potentially additive. In this review we have grouped such exposures under the working term “thermal micro-insults” to draw attention to their cumulative nature; we do not claim that TMI is an established clinical entity. Recognising cumulative low-grade heat exposure as a potentially modifiable biological factor is consistent with the broader principles of minimally invasive dentistry, and may inform the design of low-heat instrumentation, improved cooling strategies, session planning and future *in vivo* research. Prospective clinical validation, standardised sub-threshold thermal-dose descriptors and biomarker-based assessment are urgently needed before TMI can be translated into evidence-based clinical guidelines.
